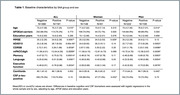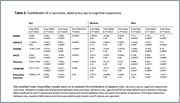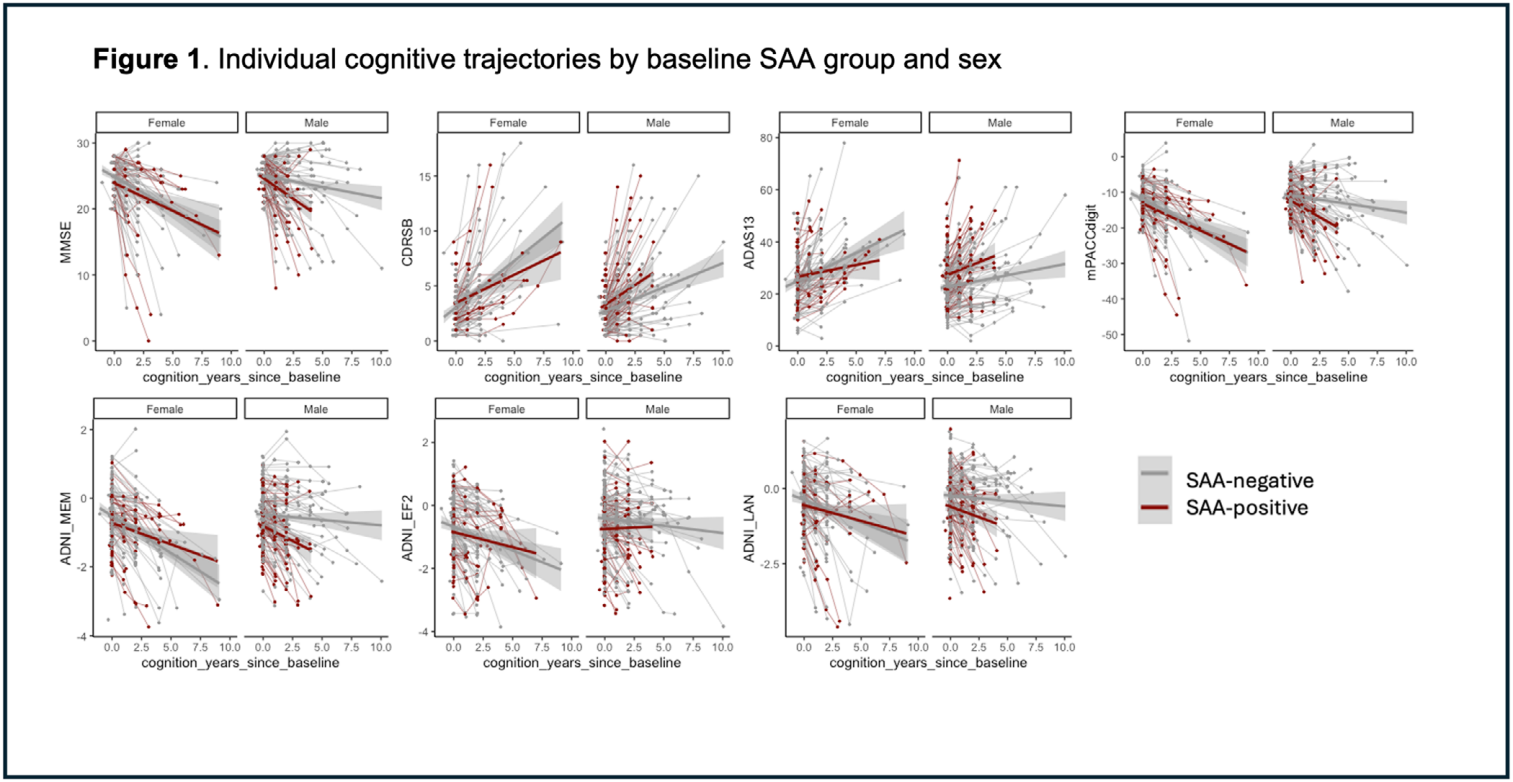# SAA‐Determined α‐Synuclein in Early AD ADNI Participants: Contribution to cognitive trajectories from a sex differences perspective

**DOI:** 10.1002/alz.095447

**Published:** 2025-01-09

**Authors:** Marta Milà‐Alomà, Zachary Hausle, Tamar M Schaap

**Affiliations:** ^1^ Department of Radiology and Biomedical Imaging, University of California, San Francisco, San Francisco, CA USA; ^2^ Department of Veterans Affairs Medical Center, Northern California Institute for Research and Education (NCIRE), San Francisco, CA USA

## Abstract

**Background:**

α‐synuclein (αSyn) aggregates are a common co‐pathology in Alzheimer’s disease (AD) and can be identified in CSF with high reliability using the Seed Amplification Assay (SAA). While AD prevalence and impact on cognition are higher in women, the opposite pattern is found for α‐synucleinopathies. Our objective was to study the independent and joint contributions of αSyn, Aß and p‐tau on cognitive trajectories in early AD individuals from a sex differences perspective.

**Method:**

We studied 529 ADNI participants (39.1% women) meeting criteria for early AD (CSF Aß+, MMSE 20‐28, and CDR 0.5 or 1.0) with baseline Aß42, p‐tau181 and αSyn determinations in CSF, and longitudinal cognition (mean follow‐up time of 5.4 years). We used logistic regressions to compare baseline cognition and biomarker levels between SAA groups in each sex. Additional models were adjusted by p‐tau181/Aß42 ratio to determine whether the impact of SAA‐positivity (SAA+) on baseline cognition was independent from the degree of AD pathology. Sex‐stratified linear mixed‐effect models were run to evaluate the contributions of baseline αSyn, Aß and p‐tau to cognitive trajectories over time. Additional models included the interaction term with sex. Age, *APOE*e4 and education were added as covariates in all models.

**Result:**

No sex differences were found in SAA+ prevalence. At baseline, p‐tau181 positivity was higher in women, particularly among SAA‐. Neither Aß burden nor cognitive scores differed by sex. SAA‐positivity at baseline was associated with higher Aß burden, particularly in men. SAA‐positivity associated with lower baseline cognitive performance in all tested cognitive domains specifically in men, and the effect was maintained after adjusting for p‐tau181/Aß42 (Table 1). Baseline SAA‐positivity also had an independent contribution to longitudinal cognitive trajectories specifically in men, with significant interactions between sex, SAA status and time in global cognition and in memory (Table 2, Figure 1).

**Conclusion:**

There are no sex differences in the prevalence of αSyn pathology in individuals with early AD. However, baseline αSyn, Aß and p‐tau181 independently associate with cognitive decline in men, but not in women. Further research is warranted to elucidate sex‐specific contributions of pathologies to cognitive decline and implications for clinical trials targeting the early AD population.